# Diisopropyl [(benzoyl­amino)­(phen­yl)meth­yl]phospho­nate

**DOI:** 10.1107/S1600536809006382

**Published:** 2009-02-28

**Authors:** Hua Fang, Mei-Juan Fang, Ying Xu, Wen-Cheng Yu, Yu-Fen Zhao

**Affiliations:** a The Third Institute of Oceanography of the State Oceanic Administration, Xiamen 361005, People’s Republic of China; b Department of Pharmaceutical Science, Medical College, Xiamen University, Xiamen 361005, People’s Republic of China; c Department of Chemistry, Key Laboratory for Chemical Biology of Fujian Province, College of Chemistry and Chemical Engineering, Xiamen University, Xiamen 361005, People’s Republic of China

## Abstract

The title compound, C_20_H_26_NO_4_P, has been obtained by the reaction of benzoyl chloride and diisoprop­yl[amino­(phen­yl)meth­yl]phospho­nate. The dihedral angle between the planes of the benzoyl­amino group and the phenyl ring is 77.0 (2)°. The crystal structure is stabilized by strong inter­molecular N—H⋯O hydrogen bonds between the doubly bonded phosphoryl O atom and the amide N atom which link the mol­ecules into pairs about a center of symmetry.

## Related literature

For the biological activity and pharmaceutical inter­est of α-hydroxy­phospho­nic acid esters, see: Stowasser *et al.* (1992 ▶); Chen *et al.* (1995 ▶). For their use as reagents in the synthesis of enol ethers and α-ketophospho­nates, see: Babak & Rahman (2001 ▶). For the synthesis, see: Drescher *et al.* (1995 ▶). For bond lengths and angles in related compunds, see: Smaardijk *et al.* (1985 ▶).
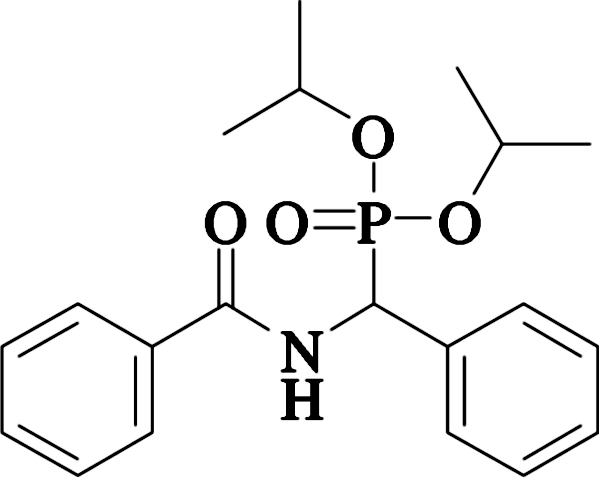


## Experimental

### 

#### Crystal data

C_20_H_26_NO_4_P
*M* *_r_* = 375.39
Triclinic, 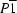

*a* = 10.839 (4) Å
*b* = 10.925 (5) Å
*c* = 11.057 (5) Å
α = 61.364 (8)°
β = 83.362 (8)°
γ = 60.470 (6)°
*V* = 987.3 (7) Å^3^
*Z* = 2
Mo *K*α radiation
μ = 0.16 mm^−1^
*T* = 273 K
0.28 × 0.21 × 0.05 mm

#### Data collection

Bruker SMART APEX area-detector diffractometer
Absorption correction: multi-scan (*SADABS*; Bruker, 2001 ▶) *T*_min_ = 0.956, *T*_max_ = 0.992
4991 measured reflections
3411 independent reflections
2509 reflections with *I* > 2σ(*I*)
*R*_int_ = 0.042

#### Refinement

*R*[*F* ^2^ > 2σ(*F* ^2^)] = 0.065
*wR*(*F* ^2^) = 0.174
*S* = 0.98
3411 reflections
235 parameters
H-atom parameters constrained
Δρ_max_ = 0.60 e Å^−3^
Δρ_min_ = −0.44 e Å^−3^

### 

Data collection: *SMART* (Bruker, 2001 ▶); cell refinement: *SAINT* (Bruker, 2001 ▶); data reduction: *SAINT*; program(s) used to solve structure: *SHELXS97* (Sheldrick, 2008 ▶); program(s) used to refine structure: *SHELXL97* (Sheldrick, 2008 ▶); molecular graphics: *ORTEP-3* (Farrugia, 1997 ▶) and *PLATON* (Spek, 2009 ▶); software used to prepare material for publication: *SHELXL97*.

## Supplementary Material

Crystal structure: contains datablocks I, global. DOI: 10.1107/S1600536809006382/fl2235sup1.cif

Structure factors: contains datablocks I. DOI: 10.1107/S1600536809006382/fl2235Isup2.hkl

Additional supplementary materials:  crystallographic information; 3D view; checkCIF report

## Figures and Tables

**Table 1:** Hydrogen-bond geometry (Å, °)

*D*—H⋯*A*	*D*—H	H⋯*A*	*D*⋯*A*	*D*—H⋯*A*
N1—H1*A*⋯O2^i^	0.86	2.05	2.895 (3)	165

Symmetry code: (i) 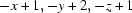.

**Fig. 1.**
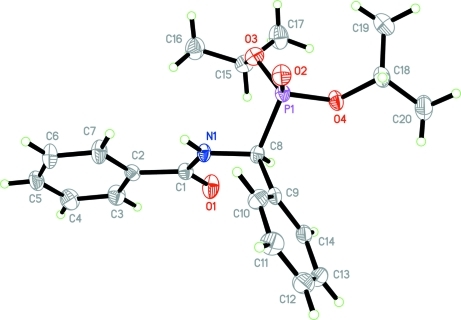
The title molecule showing the anisotropic displacement parameters of the non-hydrogen atoms at the 30% probability level. The H atoms are drawn as spheres of arbitrary radii.

**Fig. 2.**
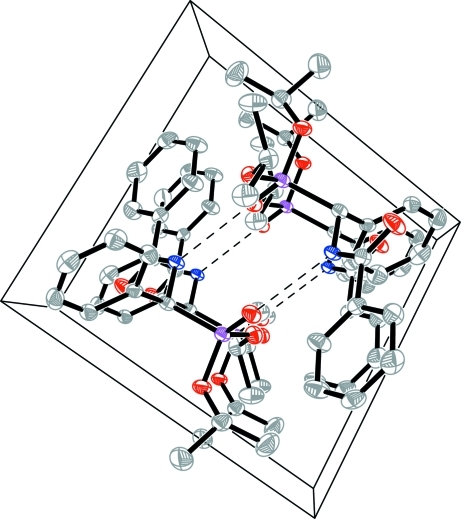
Packing diagram of title compound, showing the N—H···O hydrogen bonds as dashed lines. H atoms not involved in hydrogen bonding have been omitted. [Symmetry code: (i) -x + 1, -y + 2, -z + 1)].

**Fig. 3.**
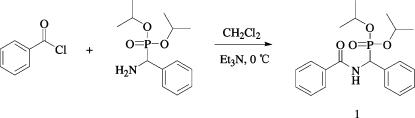
The formation of the title compound.

## supplementary crystallographic information

### Comment

In recent years α-hydroxyphosphonic acids esters have attracted much attention due to their wide biological activity (Stowasser *et al.*, 1992) and pharmaceutical interest (Chen *et al.*, 1995). They are useful reagents for the synthesis of enol ethers and α-ketophosphonates (Babak *et al.*, 2001). Bond lengths and angles in the title compound, (I), are in agreement with the values reported for related compounds (Smaardijk *et al.*, 1985). The dihedral angle between the planes of the benzoylamino group and phenyl ring is 103.0 (2)° (Fig. 1). The amide N atom is involved in a hydrogen-bonding interaction with the phosphoryl O atom of a neighboring molecule linking the molecules into pairs around a centerof symmetry (Table 1 and Fig. 2).

### Experimental

A solution of dry dichloromethane (20 ml) containing (amino-phenyl-methyl)-phosphonic acid diisopropyl ester (1 mmol, 0.27 g) and triethylamine (0.4 ml) was added dropwise to a solution of dichloromethane (10 ml) containing  benzoyl chloride (1.2 mmol, 0.17 g). The reaction mixture was stirred for 6 h at room temperature and the solvent was then removed under reduced pressure to give a residue, which was extracted with ethyl acetate (3 × 15 ml). The solution was dried over anhydrous MgSO_4_ and concentrated under vacuum to obtain a slurry residue, which was purified by silica gel column chromatography (petroleum ether/ethyl acetate = 2:1) to give  (I) as a colorless amorphous solid (Drescher, *et al.*, 1995). Single crystals of (I) suitable for X-ray analysis were obtained by slow evaporation of a petroleum ether /dichloromethane solution (1:1 *v*/*v*).

### Refinement

All H atoms were placed in geometrically idealized positions and treated as riding on their parent atoms, with C—H = 0.93 (aromatic), 0.96 (CH_3_) or 0.98 (CH), N—H = 0.86 Å and *U*_iso_(H) = 1.2*U*_eq_ (aromatic C, CH and N) or 1.5*U*_eq_ (methyl C).

### Crystal data

**Table:**

C_20_H_26_NO_4_P	*Z* = 2
*M**_r_* = 375.39	*F*(000) = 400
Triclinic, *P*1	*D*_x_ = 1.263 Mg m^−^^3^
Hall symbol: -P 1	Mo *K*α radiation, λ = 0.71073 Å
*a* = 10.839 (4) Å	Cell parameters from 1689 reflections
*b* = 10.925 (5) Å	θ = 2.3–27.7°
*c* = 11.057 (5) Å	µ = 0.16 mm^−^^1^
α = 61.364 (8)°	*T* = 273 K
β = 83.362 (8)°	Chunk, colorless
γ = 60.470 (6)°	0.28 × 0.21 × 0.05 mm
*V* = 987.3 (7) Å^3^	

### Data collection

**Table:**

Bruker APEX area-detector diffractometer	3411 independent reflections
Radiation source: fine-focus sealed tube	2509 reflections with *I* > 2σ(*I*)
graphite	*R*_int_ = 0.042
φ and ω scans	θ_max_ = 25.0°, θ_min_ = 2.1°
Absorption correction: multi-scan (*SADABS*; Bruker, 2001)	*h* = −12→12
*T*_min_ = 0.956, *T*_max_ = 0.992	*k* = −12→12
4991 measured reflections	*l* = −10→13

### Refinement

**Table:**

Refinement on *F*^2^	Primary atom site location: structure-invariant direct methods
Least-squares matrix: full	Secondary atom site location: difference Fourier map
*R*[*F*^2^ > 2σ(*F*^2^)] = 0.065	Hydrogen site location: inferred from neighbouring sites
*wR*(*F*^2^) = 0.174	H-atom parameters constrained
*S* = 0.98	*w* = 1/[σ^2^(*F*_o_^2^) + (0.097*P*)^2^] where *P* = (*F*_o_^2^ + 2*F*_c_^2^)/3
3411 reflections	(Δ/σ)_max_ < 0.001
235 parameters	Δρ_max_ = 0.60 e Å^−^^3^
0 restraints	Δρ_min_ = −0.44 e Å^−^^3^

### Special details

**Table:**

Geometry. All e.s.d.'s (except the e.s.d. in the dihedral angle between two l.s. planes) are estimated using the full covariance matrix. The cell e.s.d.'s are taken into account individually in the estimation of e.s.d.'s in distances, angles and torsion angles; correlations between e.s.d.'s in cell parameters are only used when they are defined by crystal symmetry. An approximate (isotropic) treatment of cell e.s.d.'s is used for estimating e.s.d.'s involving l.s. planes.
Refinement. Refinement of *F*^2^ against ALL reflections. The weighted *R*-factor *wR* and goodness of fit *S* are based on *F*^2^, conventional *R*-factors *R* are based on *F*, with *F* set to zero for negative *F*^2^. The threshold expression of *F*^2^ > σ(*F*^2^) is used only for calculating *R*-factors(gt) *etc*. and is not relevant to the choice of reflections for refinement. *R*-factors based on *F*^2^ are statistically about twice as large as those based on *F*, and *R*- factors based on ALL data will be even larger.

### Fractional atomic coordinates and isotropic or equivalent isotropic displacement parameters (Å^2^)

**Table:**

	*x*	*y*	*z*	*U*_iso_*/*U*_eq_	
P1	0.73254 (8)	0.85680 (9)	0.47527 (8)	0.0311 (3)	
N1	0.6453 (2)	0.6827 (3)	0.6988 (3)	0.0310 (6)	
H1A	0.5566	0.7577	0.6718	0.037*	
O1	0.8049 (2)	0.4184 (2)	0.8105 (3)	0.0546 (7)	
C1	0.6797 (3)	0.5282 (4)	0.7762 (3)	0.0339 (7)	
O2	0.6331 (2)	1.0278 (2)	0.4252 (2)	0.0412 (6)	
C2	0.5598 (3)	0.4933 (3)	0.8226 (3)	0.0336 (7)	
O3	0.6811 (2)	0.7980 (2)	0.3986 (2)	0.0400 (6)	
C3	0.5965 (4)	0.3371 (4)	0.9147 (4)	0.0505 (9)	
H3A	0.6928	0.2572	0.9424	0.061*	
O4	0.8922 (2)	0.8119 (2)	0.4564 (2)	0.0401 (6)	
C4	0.4921 (4)	0.2969 (5)	0.9671 (4)	0.0602 (11)	
H4A	0.5182	0.1906	1.0313	0.072*	
C5	0.3507 (4)	0.4130 (5)	0.9247 (4)	0.0514 (9)	
H5A	0.2804	0.3859	0.9603	0.062*	
C6	0.3123 (4)	0.5681 (4)	0.8304 (4)	0.0549 (10)	
H6A	0.2158	0.6472	0.8006	0.066*	
C7	0.4171 (3)	0.6079 (4)	0.7792 (4)	0.0505 (9)	
H7A	0.3905	0.7142	0.7142	0.061*	
C8	0.7582 (3)	0.7231 (3)	0.6607 (3)	0.0305 (7)	
H8A	0.8470	0.6220	0.6812	0.037*	
C9	0.7819 (3)	0.7826 (3)	0.7489 (3)	0.0307 (7)	
C10	0.6722 (3)	0.9170 (4)	0.7536 (3)	0.0431 (8)	
H10A	0.5844	0.9768	0.6961	0.052*	
C11	0.6917 (4)	0.9627 (4)	0.8423 (4)	0.0531 (9)	
H11A	0.6166	1.0523	0.8456	0.064*	
C12	0.8210 (4)	0.8774 (5)	0.9261 (4)	0.0562 (10)	
H12A	0.8336	0.9095	0.9856	0.067*	
C13	0.9309 (4)	0.7456 (5)	0.9221 (4)	0.0526 (9)	
H13A	1.0191	0.6879	0.9784	0.063*	
C14	0.9111 (3)	0.6979 (4)	0.8344 (3)	0.0398 (8)	
H14A	0.9862	0.6070	0.8329	0.048*	
C15	0.7424 (4)	0.6316 (4)	0.4291 (4)	0.0435 (8)	
H15A	0.7990	0.5581	0.5230	0.052*	
C16	0.6184 (4)	0.6079 (5)	0.4248 (4)	0.0608 (10)	
H16A	0.5615	0.6255	0.4942	0.091*	
H16B	0.6538	0.5000	0.4432	0.091*	
H16C	0.5604	0.6832	0.3342	0.091*	
C17	0.8383 (4)	0.6056 (5)	0.3243 (4)	0.0635 (11)	
H17A	0.9161	0.6209	0.3328	0.095*	
H17B	0.7841	0.6815	0.2319	0.095*	
H17C	0.8758	0.4979	0.3409	0.095*	
C18	0.9332 (4)	0.9289 (4)	0.3580 (4)	0.0458 (8)	
H18A	0.8731	1.0307	0.3593	0.055*	
C19	0.9101 (5)	0.9581 (6)	0.2149 (4)	0.0810 (14)	
H19A	0.8101	1.0015	0.1875	0.121*	
H19B	0.9658	0.8583	0.2131	0.121*	
H19C	0.9393	1.0334	0.1513	0.121*	
C20	1.0845 (4)	0.8639 (6)	0.4093 (5)	0.0816 (14)	
H20A	1.0916	0.8510	0.5009	0.122*	
H20B	1.1162	0.9373	0.3470	0.122*	
H20C	1.1438	0.7612	0.4133	0.122*	

### Atomic displacement parameters (Å^2^)

**Table:**

	*U*^11^	*U*^22^	*U*^33^	*U*^12^	*U*^13^	*U*^23^
P1	0.0254 (4)	0.0278 (5)	0.0360 (5)	−0.0115 (3)	0.0062 (3)	−0.0149 (4)
N1	0.0210 (12)	0.0262 (13)	0.0398 (14)	−0.0105 (10)	0.0066 (11)	−0.0139 (12)
O1	0.0308 (12)	0.0297 (13)	0.0752 (18)	−0.0100 (10)	0.0017 (12)	−0.0102 (13)
C1	0.0348 (17)	0.0299 (17)	0.0363 (17)	−0.0170 (14)	0.0064 (14)	−0.0150 (15)
O2	0.0342 (11)	0.0288 (12)	0.0497 (13)	−0.0118 (9)	0.0050 (10)	−0.0155 (11)
C2	0.0407 (17)	0.0328 (17)	0.0343 (17)	−0.0228 (15)	0.0099 (14)	−0.0176 (15)
O3	0.0379 (12)	0.0357 (12)	0.0442 (12)	−0.0141 (10)	0.0030 (10)	−0.0217 (11)
C3	0.049 (2)	0.036 (2)	0.054 (2)	−0.0231 (17)	0.0061 (18)	−0.0111 (18)
O4	0.0284 (11)	0.0367 (12)	0.0448 (13)	−0.0162 (10)	0.0120 (10)	−0.0142 (11)
C4	0.075 (3)	0.050 (2)	0.055 (2)	−0.046 (2)	0.011 (2)	−0.011 (2)
C5	0.059 (2)	0.073 (3)	0.052 (2)	−0.051 (2)	0.0245 (19)	−0.035 (2)
C6	0.0391 (19)	0.055 (2)	0.077 (3)	−0.0298 (18)	0.0200 (19)	−0.032 (2)
C7	0.0383 (18)	0.0377 (19)	0.070 (2)	−0.0226 (16)	0.0128 (18)	−0.0197 (19)
C8	0.0236 (14)	0.0258 (16)	0.0400 (17)	−0.0107 (12)	0.0052 (13)	−0.0164 (15)
C9	0.0266 (15)	0.0321 (17)	0.0336 (16)	−0.0164 (13)	0.0097 (13)	−0.0155 (15)
C10	0.0386 (18)	0.0392 (19)	0.0472 (19)	−0.0152 (15)	0.0013 (16)	−0.0216 (17)
C11	0.057 (2)	0.050 (2)	0.059 (2)	−0.0245 (19)	0.012 (2)	−0.035 (2)
C12	0.080 (3)	0.068 (3)	0.052 (2)	−0.053 (2)	0.019 (2)	−0.036 (2)
C13	0.052 (2)	0.066 (2)	0.044 (2)	−0.040 (2)	0.0010 (18)	−0.018 (2)
C14	0.0338 (17)	0.0424 (19)	0.0400 (17)	−0.0211 (15)	0.0077 (15)	−0.0163 (16)
C15	0.0454 (19)	0.0363 (18)	0.048 (2)	−0.0177 (15)	0.0026 (17)	−0.0213 (17)
C16	0.064 (2)	0.068 (3)	0.072 (3)	−0.041 (2)	0.019 (2)	−0.043 (2)
C17	0.058 (2)	0.068 (3)	0.082 (3)	−0.033 (2)	0.033 (2)	−0.052 (3)
C18	0.0456 (19)	0.044 (2)	0.049 (2)	−0.0285 (17)	0.0128 (17)	−0.0182 (18)
C19	0.095 (3)	0.108 (4)	0.050 (2)	−0.073 (3)	0.020 (2)	−0.023 (3)
C20	0.063 (3)	0.094 (3)	0.074 (3)	−0.056 (3)	0.006 (2)	−0.013 (3)

### Geometric parameters (Å, °)

**Table:**

P1—O2	1.456 (2)	C10—H10A	0.9300
P1—O3	1.559 (2)	C11—C12	1.369 (5)
P1—O4	1.567 (2)	C11—H11A	0.9300
P1—C8	1.809 (3)	C12—C13	1.361 (5)
N1—C1	1.337 (4)	C12—H12A	0.9300
N1—C8	1.451 (4)	C13—C14	1.379 (5)
N1—H1A	0.8600	C13—H13A	0.9300
O1—C1	1.226 (3)	C14—H14A	0.9300
C1—C2	1.498 (4)	C15—C16	1.498 (5)
C2—C3	1.365 (4)	C15—C17	1.500 (5)
C2—C7	1.370 (4)	C15—H15A	0.9800
O3—C15	1.460 (4)	C16—H16A	0.9600
C3—C4	1.380 (5)	C16—H16B	0.9600
C3—H3A	0.9300	C16—H16C	0.9600
O4—C18	1.460 (4)	C17—H17A	0.9600
C4—C5	1.363 (5)	C17—H17B	0.9600
C4—H4A	0.9300	C17—H17C	0.9600
C5—C6	1.358 (5)	C18—C20	1.482 (5)
C5—H5A	0.9300	C18—C19	1.483 (5)
C6—C7	1.380 (5)	C18—H18A	0.9800
C6—H6A	0.9300	C19—H19A	0.9600
C7—H7A	0.9300	C19—H19B	0.9600
C8—C9	1.507 (4)	C19—H19C	0.9600
C8—H8A	0.9800	C20—H20A	0.9600
C9—C14	1.378 (4)	C20—H20B	0.9600
C9—C10	1.382 (4)	C20—H20C	0.9600
C10—C11	1.370 (4)		
			
O2—P1—O3	109.31 (12)	C10—C11—H11A	119.7
O2—P1—O4	114.66 (12)	C13—C12—C11	119.9 (3)
O3—P1—O4	108.59 (12)	C13—C12—H12A	120.0
O2—P1—C8	116.66 (13)	C11—C12—H12A	120.0
O3—P1—C8	107.29 (13)	C12—C13—C14	119.7 (3)
O4—P1—C8	99.67 (12)	C12—C13—H13A	120.1
C1—N1—C8	119.7 (2)	C14—C13—H13A	120.1
C1—N1—H1A	120.1	C9—C14—C13	121.2 (3)
C8—N1—H1A	120.1	C9—C14—H14A	119.4
O1—C1—N1	121.6 (3)	C13—C14—H14A	119.4
O1—C1—C2	120.7 (3)	O3—C15—C16	106.6 (3)
N1—C1—C2	117.7 (3)	O3—C15—C17	108.8 (3)
C3—C2—C7	118.6 (3)	C16—C15—C17	113.3 (3)
C3—C2—C1	117.3 (3)	O3—C15—H15A	109.4
C7—C2—C1	124.1 (3)	C16—C15—H15A	109.4
C15—O3—P1	126.33 (19)	C17—C15—H15A	109.4
C2—C3—C4	120.6 (3)	C15—C16—H16A	109.5
C2—C3—H3A	119.7	C15—C16—H16B	109.5
C4—C3—H3A	119.7	H16A—C16—H16B	109.5
C18—O4—P1	123.20 (19)	C15—C16—H16C	109.5
C5—C4—C3	120.0 (3)	H16A—C16—H16C	109.5
C5—C4—H4A	120.0	H16B—C16—H16C	109.5
C3—C4—H4A	120.0	C15—C17—H17A	109.5
C6—C5—C4	120.1 (3)	C15—C17—H17B	109.5
C6—C5—H5A	119.9	H17A—C17—H17B	109.5
C4—C5—H5A	119.9	C15—C17—H17C	109.5
C5—C6—C7	119.7 (3)	H17A—C17—H17C	109.5
C5—C6—H6A	120.2	H17B—C17—H17C	109.5
C7—C6—H6A	120.2	O4—C18—C20	106.7 (3)
C2—C7—C6	121.0 (3)	O4—C18—C19	110.1 (3)
C2—C7—H7A	119.5	C20—C18—C19	113.7 (4)
C6—C7—H7A	119.5	O4—C18—H18A	108.7
N1—C8—C9	112.6 (2)	C20—C18—H18A	108.7
N1—C8—P1	112.04 (18)	C19—C18—H18A	108.7
C9—C8—P1	113.18 (19)	C18—C19—H19A	109.5
N1—C8—H8A	106.1	C18—C19—H19B	109.5
C9—C8—H8A	106.1	H19A—C19—H19B	109.5
P1—C8—H8A	106.1	C18—C19—H19C	109.5
C14—C9—C10	118.1 (3)	H19A—C19—H19C	109.5
C14—C9—C8	120.7 (3)	H19B—C19—H19C	109.5
C10—C9—C8	121.0 (2)	C18—C20—H20A	109.5
C11—C10—C9	120.5 (3)	C18—C20—H20B	109.5
C11—C10—H10A	119.7	H20A—C20—H20B	109.5
C9—C10—H10A	119.7	C18—C20—H20C	109.5
C12—C11—C10	120.5 (3)	H20A—C20—H20C	109.5
C12—C11—H11A	119.7	H20B—C20—H20C	109.5
			
C8—N1—C1—O1	−3.5 (4)	O2—P1—C8—N1	82.0 (2)
C8—N1—C1—C2	175.2 (2)	O3—P1—C8—N1	−40.9 (2)
O1—C1—C2—C3	6.2 (4)	O4—P1—C8—N1	−153.99 (19)
N1—C1—C2—C3	−172.5 (3)	O2—P1—C8—C9	−46.6 (2)
O1—C1—C2—C7	−174.3 (3)	O3—P1—C8—C9	−169.52 (19)
N1—C1—C2—C7	7.0 (4)	O4—P1—C8—C9	77.4 (2)
O2—P1—O3—C15	−173.2 (2)	N1—C8—C9—C14	116.7 (3)
O4—P1—O3—C15	61.0 (3)	P1—C8—C9—C14	−115.0 (3)
C8—P1—O3—C15	−45.8 (3)	N1—C8—C9—C10	−58.9 (4)
C7—C2—C3—C4	−2.5 (5)	P1—C8—C9—C10	69.4 (3)
C1—C2—C3—C4	177.0 (3)	C14—C9—C10—C11	−0.7 (5)
O2—P1—O4—C18	−19.8 (3)	C8—C9—C10—C11	174.9 (3)
O3—P1—O4—C18	102.8 (2)	C9—C10—C11—C12	1.0 (5)
C8—P1—O4—C18	−145.1 (2)	C10—C11—C12—C13	−0.4 (6)
C2—C3—C4—C5	1.4 (6)	C11—C12—C13—C14	−0.5 (6)
C3—C4—C5—C6	0.3 (6)	C10—C9—C14—C13	−0.2 (5)
C4—C5—C6—C7	−0.8 (5)	C8—C9—C14—C13	−175.8 (3)
C3—C2—C7—C6	2.0 (5)	C12—C13—C14—C9	0.8 (5)
C1—C2—C7—C6	−177.4 (3)	P1—O3—C15—C16	136.8 (3)
C5—C6—C7—C2	−0.4 (6)	P1—O3—C15—C17	−100.8 (3)
C1—N1—C8—C9	−101.9 (3)	P1—O4—C18—C20	156.8 (3)
C1—N1—C8—P1	129.2 (2)	P1—O4—C18—C19	−79.4 (3)

### Hydrogen-bond geometry (Å, °)

**Table:**

*D*—H···*A*	*D*—H	H···*A*	*D*···*A*	*D*—H···*A*
N1—H1A···O2^i^	0.86	2.05	2.895 (3)	165

Symmetry codes: (i) −*x*+1, −*y*+2, −*z*+1.

## References

[bb1] Babak, K. & Rahman, N. (2001). *Synth. Commun.* **31**, 2245–2250.

[bb2] Bruker (2001). *SAINT*, *SMART* and *SADABS* Bruker AXS Inc., Madison, Wisconsin, USA.

[bb3] Chen, R., Liu, L. & Zhang, Z. (1995). *Heteroatom. Chem.* **6**, 503–506.

[bb4] Drescher, M., Hammerschmidt, F. & Kählig, H. (1995). *Synthesis*, **10**, 1267–1272.

[bb5] Farrugia, L. J. (1997). *J. Appl. Cryst.* **30**, 565.

[bb6] Sheldrick, G. M. (2008). *Acta Cryst.* A**64**, 112–122. 10.1107/S0108767307043930 18156677

[bb7] Smaardijk, Ab. A., Noorda, S., van Bolhuis, F. & Wynberg, H. (1985). *Tetrahedron Lett.* **26**, 493–496.

[bb8] Spek, A. L. (2009). *Acta Cryst.* D**65**, 148–155. 10.1107/S090744490804362X PMC2631630 19171970

[bb9] Stowasser, B., Budt, K.-H., Jian-Qi, L., Peyman, A. & Ruppert, D. (1992). *Tetrahedron Lett.* **33**, 6625–6628.

